# Drug-Induced Mitochondrial Toxicity in the Geriatric Population: Challenges and Future Directions

**DOI:** 10.3390/biology8020032

**Published:** 2019-05-11

**Authors:** Yvonne Will, Jefry E. Shields, Kendall B. Wallace

**Affiliations:** 1 SCIENTIA LLC, Ledyard, CT 06339, USA; jefshields@yahoo.ca; 2 Department of Biomedical Sciences, University of Minnesota Medical School, Duluth, MN 55455, USA; kwallace@d.umn.edu

**Keywords:** drug-induced mitochondrial toxicity, polypharmacy, aging

## Abstract

Mitochondrial function declines with age, leading to a variety of age-related diseases (metabolic, central nervous system-related, cancer, etc.) and medication usage increases with age due to the increase in diseases. Drug-induced mitochondrial toxicity has been described for many different drug classes and can lead to liver, muscle, kidney and central nervous system injury and, in rare cases, to death. Many of the most prescribed medications in the geriatric population carry mitochondrial liabilities. We have demonstrated that, over the past decade, each class of drugs that demonstrated mitochondrial toxicity contained drugs with both more and less adverse effects on mitochondria. As patient treatment is often essential, we suggest using medication(s) with the best safety profile and the avoidance of concurrent usage of multiple medications that carry mitochondrial liabilities. In addition, we also recommend lifestyle changes to further improve one’s mitochondrial function, such as weight loss, exercise and nutrition.

## 1. Introduction

Known as the powerhouse of the cell, mitochondria are recognized for their ability to produce large amounts of a cell’s energy currency, ATP (Adenosine Triphosphate), as well as being the only other organelle that contains DNA apart from the nucleus. Virtually all eukaryotic cells employ mitochondria to produce the majority of ATP needed to perform cellular functions (e.g., replication, protein synthesis, muscle contraction, etc.) and maintain cellular viability. The electron transport chain (ETC) and ATP synthase are interdependent systems in the mitochondria that generate ATP by coupling the electrochemical potential across the inner mitochondrial membrane created by the ETC to the phosphorylation of ADP by ATP synthase. The protein complexes comprising the ETC shuttle electrons down an energy gradient to ultimately reduce molecular oxygen to water, driving oxidative phosphorylation for ATP synthesis. Compromising the function of any of these protein complexes reduces the efficiency of ATP production and endangers the cell’s ability to perform its function and can lead to cell death. Additionally, uncoupling the membrane potential from ATP synthesis through the loss of the inner mitochondrial membrane impermeability dissipates the harvested energy as heat and bypasses ATP synthesis and greatly impairs the cell’s viability. Mitochondria contain their own DNA (mtDNA) and both inherited and acquired mutations in mtDNA have been linked to well over 100 mitochondrial syndromes such as Leber’s hereditary optic neuropathy, mitochondrial myopathy, encephalomyopathy, lactic acidosis, stroke-like symptoms and myoclonic epilepsy with ragged red fibers [1]. MtDNA is susceptible to oxidative stress due to its proximity to the ETC and the fact that it does not contain histones or a repair mechanism.

**Figure 1:**
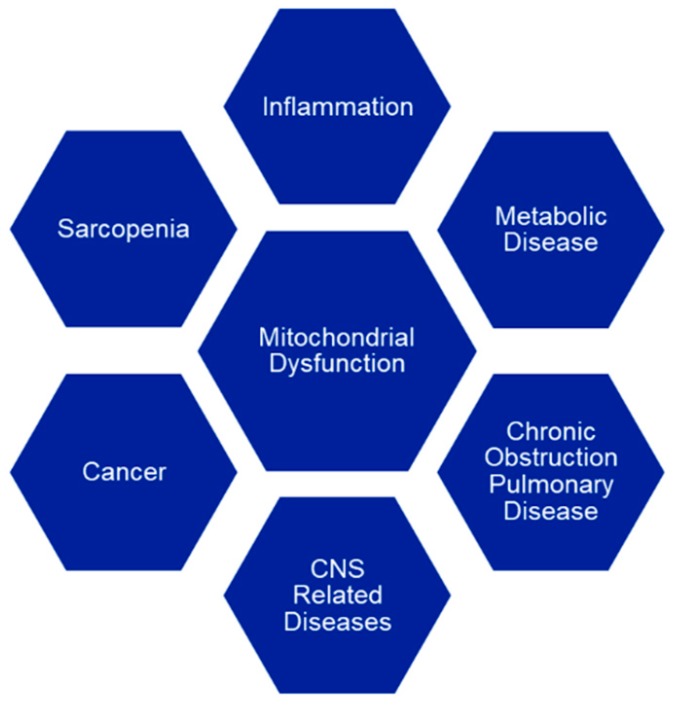
Mitochondrial dysfunction is implicated in many age-related diseases such as metabolic diseases (T2DM, obesity, cardiovascular and cerebrovascular disease, Non-Alcoholic Fatty Liver Disease (NAFDL)) [2,3,4,5,6,7], CNS-related diseases (Parkinson’s, Alzheimer’s and Huntington’s disease, hearing loss, cataracts) [8,9,10,11], inflammation (osteoarthritis) [12], cancer [13,14], sarcopenia [15] and chronic obstructive pulmonary disease (COPD) [16].

As we age, mitochondrial function declines and it is described as the “mitochondrial theory of aging”, leading to a variety of age-related diseases (Figure 1). 

These age-related diseases are often treated with multiple medications, some of which are known to cause drug-induced mitochondrial toxicity. In this review, we postulate that drug-induced mitochondrial dysfunction can increase in frequency and severity in the geriatric population due to two independent factors associated with aging, compromised mitochondrial function and polypharmacy. 

## 2. The Mitochondrial Theory of Aging

The “Mitochondrial Theory of Aging” has gained considerable prominence in discussions of longevity, inherent vitality and the biology of mortality. First proposed in his book published in 1928, Raymond Pearl provided a lengthy account of the evidence leading him to posit that longevity is inversely proportional to “the rate of living”; by that, he is referring to the expenditure of energy. His theory is that one is born with a finite quantum of energy, the expenditure of which defines his or her mortality [17]. Although the tenants of this association may hold well for drosophila and perhaps other poikilotherms, in mammals this theory is essentially the antithesis for vitality. There is an abundance of evidence, much of which is current and clinical, demonstrating that energy expenditure is actually vital to delaying mortality and extending one’s longevity [18,19,20]. This is all summarized in the pronouncements that exercise and diet are fundamental to a long and healthy life, perhaps delaying the natural biological regression that is associated with aging.

It is a well-established fact that mitochondrial numbers, function and bioenergetic capacity decline progressively with age [21,22]; Harman summarized this when he coined the term “Mitochondrial Clock” in 1972 [23], which is rooted in his seminal thesis dating from 1956 on the free radical theory of aging [24,25]. This theory is based on the demonstrated accumulation of oxidative damage to lipid, protein, and nucleic acids in aged tissues [26], which Harman attributed to the increased free radical generation observed with aging. The thought is that as biochemical efficiency and physiological function secede with age, the rate of free radical generation increases, leading to increased levels of oxidative damage. This line of thinking continues by prospecting that increased oxidative damage to the ETC within the mitochondrial compartment leads to a progressive loss of coupling efficiency and increased rates of free radical liberation from the electron transport chain. Accordingly, rather than being the consequence of aging, mitochondria are also implicated as the progenitor of the aging process [25]. It becomes quite apparent that the “Mitochondrial Theory of Aging” and the “Free Radical Theory of Aging” are closely intertwined, and it is subject to debate as to which is the cause and which is the consequence of aging (the chicken vs. egg conundrum). 

Regardless of whether it is the cause or consequence, hallmarks of mitochondrial aging [19] include a decrease in the number and an increase in the size of mitochondria [27], a net decrease in the cellular mitochondrial membrane potential, a decreased ADP:O ratio (oxidative phosphorylation) and respiratory control index (RCI), and a loss of individual ETC enzyme activities [21,22]; the steady-state [ATP] concentration, however, tends to be unaffected. 

Although each of these factors can individually contribute to the progressive loss of mitochondrial function with age, it is far more plausible that they reflect a much broader and concerted decline in homeostatic regulation directed from a molecular level [28]. Molecular hallmarks associated with mitochondrial aging include a decreased mtDNA copy number [29] accompanied by the accumulation of mutations to the mitochondrial genome [30,31], reduced abundance of both nuclear and mitochondrial encoded mRNA [22,32], decreased mitochondrial protein synthesis [33,34,35,36], and the dysregulation of the homeostatic balance between mitochondrial biogenesis, fission and mitophagy [19,28].

Ristow and Zarse [20] were first to apply the term “mitohormesis” to conceptualize the loss of the adaptive response of mitochondria by means of regulating mitochondrial homeostasis, contributing to cell and organismal senescence. This has been followed by a number of reviews suggesting that the age-related accumulation of defective mitochondria, accompanied by the loss of ability to clear these from the cell by way of mitophagy and replace them with healthy mitochondria through mitochondrial fission and biogenesis, underpins the mitochondrial theory of aging [19,28,37,38,39]. This loss of hormesis or mitochondrial homeostasis is equated to a progressive decline in the processes devoted to quality control (QC^mt^) to sustain mitochondrial fidelity and capacity within the aging cell [19,28,40].

**Figure 2:**
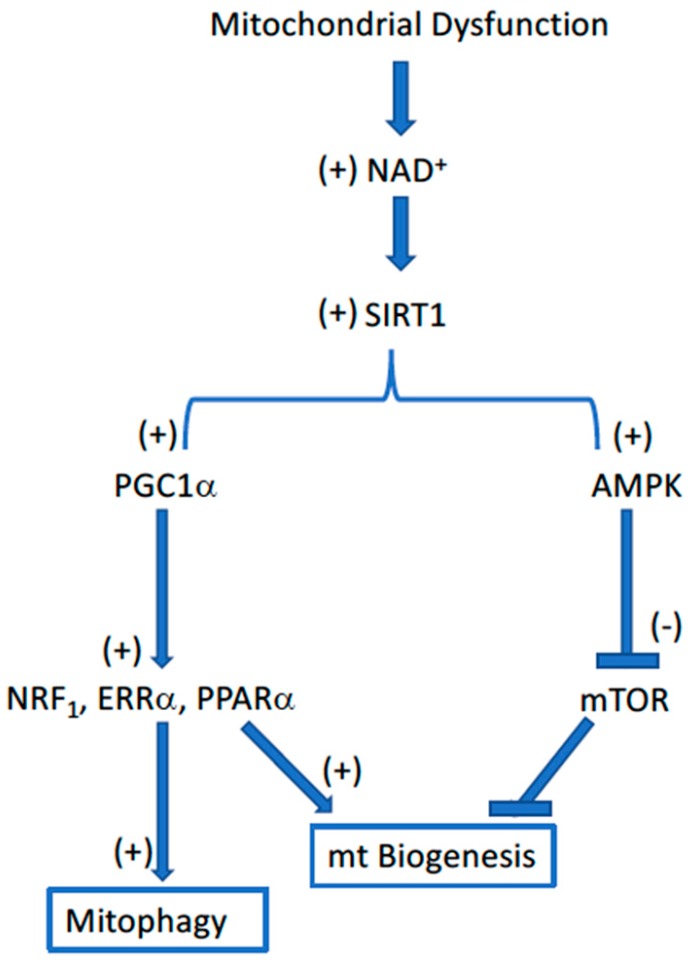
Flow diagram illustrating the interrelationships governing mitochondrial homeostasis in response to the loss of mitochondrial function, such as that which occurs with aging. The Sirt1-dependent regulation of both PGC1α and AMPK provides a well-controlled integration of the disposal of dysfunctional mitochondria (mitophagy) and their replacement with new, supposedly fully functional, mitochondria (biogenesis).

Two major surveillance mechanisms exist within the cell for sensing dysfunctional mitochondria: (1) bioenergetic or nutrient signaling and (2) mitochondrial proteostasis [19,28]. Nutrient sensing is largely by way of activating AMP kinase (AMPK), SIRT1, and the sestrins for a negative nutrient balance, and mTOR for nutrient surplus (Figure 2).

Pyridine nucleotide redox status (NAD^+^/NADH) is a key factor linking the nutritional status of the cell to both free radical generation and mitochondrial homeostasis. Nutritional deficiency (i.e., mitochondrial dysfunction) is associated with a high NAD^+^/NADH ratio and low rates of free radical generation. Under these same conditions, the accumulation of high [NAD^+^] is also associated with high rates of mitochondrial proliferation (biogenesis) and disposal of defective mitochondria (mitophagy), both of which occur by way of the NAD^+^-dependent activation of SIRT1. The activation of SIRT1 by NAD^+^ stimulates mitochondrial biogenesis, both by stimulating the PGC1α-dependent activation of mitochondrial biogenesis combined with the AMPK-dependent inhibition of the negative regulator mTOR. The second surveillance pathway, mitochondrial proteostasis, actuates the unfolded protein response (UPR^mt^) indigenous to mitochondria, where AMPK and mTOR are competing regulators. Collectively, these nutrient sensor pathways regulate both the disposal of defective mitochondria and their replenishment with supposedly fully functional mitochondria. 

There is growing evidence that these various mitochondrial signaling pathways are compromised with aging. It may be as simple as the age-associated decrease of [NAD^+^] [41,42] that is responsible for the lower rates of both mitochondrial biogenesis and mitophagy in aged tissues. Likewise, AMPK and the sirtuins are down-regulated with aging [43,44,45] as are the concentrations of PGC1α and the rate of mitophagic elimination of dysfunctional mitochondria [46]. These changes suggest loss of molecular mechanisms regulating quality control as being responsible for the ineffective nutrient sensing of mitochondria in aging tissues.

Regardless of the cause, the bioenergetic phenotype of mitochondria from most tissues of aged individuals is very different from that of young adults, which may be a significant factor accounting for the higher rates of drug-induced adverse events in geriatric populations. There is growing appreciation of the possibility that the decline in molecular regulation of mitochondrial hormesis is the primary factor that impedes the ability of aging mitochondria to withstand or adapt to external inputs [26,28,47]. It may be this loss of homeostatic regulation that underlies the sometimes-greater susceptibility of elderly patients to drug-induced adverse events.

## 3. Drug-Induced Mitochondrial Toxicity

Drug-induced mitochondrial toxicity has been studied in academic settings for well over 50 years. Indeed, many such studies relied on xenobiotic inhibitors, such as rotenone, antimycin and oligomycin, to deduce the function of the ETC, and uncouplers such as 2,4 dinitrophenol, to understand how mitochondria generate ATP. As a result, we should not be surprised that pharmaceutical xenobiotics intended to be therapeutics could also have deleterious ‘side effects’ on mitochondrial function. Drugs can inhibit mitochondrial function in many different ways such as through the inhibition of ETC protein complexes, inhibition of ATP synthase, inhibition of enzymes of the citric acid cycle, inhibition of various mitochondrial transporters, inhibition of the mitochondrial transcription and translational machinery, as well as through the uncoupling of the ETC from ATP synthase. However, most of these side effects were not detected in preclinical animal studies. This is due to the fact that in vivo toxicity studies are usually done in drug-naïve, young adult animals that have robust mitochondrial reserves; lack of sufficient genetic diversity to allow for idiosyncratic responses; absence of environmental factors; co-medication; insensitivity of histopathology for revealing mitochondrial failure. Much progress has been made in the past decade to develop a variety of high-throughput applicable organelle based and in vitro cell models preclinically [48]. Whereas, traditionally, cells were cultured in high glucose and were unresponsive to mitochondrial toxicity due to shifting to glycolysis for energy production, recently developed cell models grown in galactose can correctly identify potential drug-induced mitochondrial toxicity with greater sensitivity [49,50]. Drugs can also now be tested for potentially causing mitochondrial toxicity in 96- and 384-well formats using solid and soluble oxygen sensors [51,52].

Drug-induced mitochondrial toxicity has been recognized to cause organ toxicity to the liver, skeletal muscle, kidney, heart and the central nervous system. Drug classes identified to cause mitochondrial toxicity are anti-diabetic drugs (thiazolidinediones, fibrates, biguanides), cholesterol lowering drugs (statins), anti-depressants (SARIs), pain medications (NSAIDs), certain antibiotics (fluroquinolones, macrolide), and anti-cancer drugs (kinase inhibitors and anthracyclins) [48]. Most of these observations have been made through studies in isolated mitochondria and cell lines [48].

Over the past decade, our laboratories examined the effects of many different drug classes and found that in each drug class, some members of the class displayed greater potency for adverse effects in vitro than others and the rank order of toxicity observed mimicked the safety profile reported in patients. For example, the anti-diabetic drug Resulin (triglitazone), which was discontinued from the market for liver toxicity, caused greater mitochondrial toxicity when tested in isolated mitochondria than Actos (pioglitazone), which is on the market with a much better safety profile [53]. The same is true for the anti-depressant Zerzone (nefazadone), which belongs to the class of serotonin antagonist reuptake inhibitors. Whereas Zerzone was attrited due to liver toxicity caused at least in part by mitochondrial toxicity, Buspar (buspirone) is still on the market and is well tolerated [54]. Another class of anti-diabetic drugs is the biguanides. Phenformin was discontinued because it caused death by lactic acidosis which is considered a hallmark of mitochondrial toxicity. Glucophage (metformin), which causes much less mitochondrial toxicity is widely used in the clinic and only in rare occasions causes lactic acidosis in most likely already predisposed patients [55]. Table 1 provides additional examples of drugs and drug classes studied by our labs.

## 4. Polypharmacy in the Geriatric Population

Geriatric patients are not only predisposed by having lower mitochondrial function due to age but are often also experiencing one or more of the age-related diseases mentioned above (Figure 1). In addition, they are also often taking multiple medications (polypharmacy). In the United States, a 2010 and 2011 survey found that 87% of a representative sampling of 2206 adults aged 62 through 85, used at least one prescription medication and that more than one-third of the group were taking five or more prescription medications [62]. Additionally, it was found that 38% of those surveyed were using over-the-counter medications. A further study by Saraf et al. showed that following acute illness or injury, an average of 14 prescriptions were given to geriatric patients discharged from hospitals to skilled nursing facilities and that over one-third of these prescriptions included side-effects that could aggravate underlying geriatric conditions [63].

Of the most commonly used prescription and over-the-counter drugs in the US for older adults [64], many are known to cause mitochondrial toxicity such as the cholesterol lowering drugs (Zocor, Lipitor, Pravacol, Crestor), pain medication (Aspirin, Tylenol, Aleve) and heartburn medication (Prilosec). Table 2 lists references for mitochondrial dysfunction reported for these prescription and over-the-counter (OTC) medications.

It is important to note that most studies have been conducted using isolated mitochondria and cell systems and often these systems are not of human nature. Also, it is important to understand that the safety margin for a particular drug will depend on exposure (Cmax) [54,97] as well as other contributing mechanistic toxicities [98]. For example, Trovan and Serzone (troglitazone and nefazodone) not only have inhibitory effects on mitochondrial function, but also cause additional toxicities through inhibition of the bile salt efflux pump (BSEP) and the formation of reactive metabolites.

Cholesterol lowering statins are the most commonly prescribed drugs in the geriatric population. Almost 50% of the geriatric population was taking a statin in 2011–2014 versus only 20% in 1999–2002. Statins can cause myopathy in 10–15% of the patients. The adverse events range from mild myalgia and fatigue, to life threatening rhabdomyolysis. Baycol was removed from the market in 2001 because it caused death in 52 patients by rhabdomyolysis, which led to kidney failure [99]. Women, frail individuals and those with low body index as well as patients with increased alcohol consumption are at higher risk. Harper and Jacobson, 2010, noted that Zocor (simvastatin) has greater muscle toxicity and drug interactions and should be avoided if the patient has had adverse events and should be substituted with Lescol (fluvastatin) or Crestor (rosuvastatin) [100].

Mitochondrial toxicity has been postulated as a contributing, if not causal, factor of the observed muscle toxicity. It has been postulated that a deficit in CoQ10 is the cause. CoQ10 is an essential electron carrier in the ETC and the pathway for its synthesis has commonality with that of the cholesterol pathway. A decrease in circulatory CoQ10 levels of 27–50% has been reported [101]. However, since circulating CoQ10 is carried by low-density lipoproteins (LDL) the observed decrease may simply reflect the decrease in circulating levels of LDL [101]. Very few studies have actually examined the level of CoQ10 in muscle, probably due to the invasive nature of human muscle biopsies. While these studies did demonstrate significant decreases (30%) in muscle CoQ10 levels, they failed to demonstrate a decrease in ATP synthesis and no myopathic side effects were reported [102,103]. Since muscle toxicity seems to be patient specific and somewhat rare (the same is true for liver toxicity caused by troglitazone and nefazodone), it is most likely that patients will have either underlying mitochondrial dysfunction (for example, a silent mitochondrial disease) or have different levels of transporter expression (MCT4 in the case of statin accumulation) due to polymorphism in enzymes involved in statin metabolism [104].

Beyond the effect on CoQ10, statins have been reported to have direct effects on the ETC, especially the lipophilic statins, simvastatin and fluvastatin. In addition, statins have the ability to uncouple oxidative phosphorylation, inhibit fatty acid beta oxidation, induce mitochondrial permeability transition, and cause oxidative stress and decrease mtDNA levels by thus far unidentified mechanisms [105]. 

It has been reported that elderly patients taking cholesterol lowering drugs (Zocor, Lipitor, etc.) have shown drug–drug interactions (DDI), particularly with the anti-lipidemic drug Lopid (gemfibrozil [53,106]), but also with certain antibiotic classes, such as macrolides [107,108] and fluoroquinolones [109,110] and the heart medication, Nexteron (amiodarone) [64,111]. All of these drugs have been shown to cause mitochondrial toxicity by targeting a variety of, as well as multiple, mitochondrial mechanisms/targets.

While it is well documented that this is mainly due to changes in the drug metabolizing function in elderly patients, we hypothesize that polypharmacy with multiple drugs affecting mitochondrial function could contribute to the toxicities observed. We already spoke of the general mitochondrial decay with age and in age-related diseases.

How do we imagine an individual’s lifestyle affects their mitochondrial health and energy production, or their response to medications that carry a potential mitochondrial liability (Table 3)? Differences in body mass index (BMI) have shown that obesity correlates with mitochondrial dysfunction [112]. Additionally, diets of high fat and high sucrose have been shown to be detrimental to mitochondrial function [113,114]. Mitochondria are also weakened by inactive lifestyles and can be revitalized with exercise [115,116]. Studies by DiNicolantonio et al. show deleterious effects on mitochondrial function when consuming large quantities of sugar-containing foods and drinks [117], while other labs have shown that imbibing red wine in moderation [118] and drinking green tea [119] have an advantageous effect on mitochondrial function. Even the choice of area of residency can lead to mitochondrial impairment. Lim et al. have studied the effects of incidental ingestion of pesticides/herbicides and have shown a correlation between the area of residence and increased exposure to exogenous agricultural chemicals which have adverse effects on mitochondrial function [120].

Now, we imagine how different individuals with ideal vs. less than ideal lifestyles might tolerate mitochondrial insults from medications. “Patient 1” has a lifestyle that promotes mitochondrial health while “Patient 2’s” lifestyle predisposes them to mitochondrial decay and toxicity in a number of ways. Taking medications that impair mitochondria is far more concerning for Patient 2 than for Patient 1. Patient 2’s healthcare providers could examine their lifestyle and medication regiment and act in unison to come up with a medication plan that minimizes mitochondrial liabilities with the drugs they are prescribing for them, opting for drugs with the best safety profile. As mentioned previously, the combination of Zocor with Lopid should at least be substituted with Crestor. An improvement in Patient 2’s diet could eliminate the daily use of Prilosec and Patient 2’s pain medication of choice should be Aspirin or Tylenol in moderation.

The question arises if mitochondrial dysfunction/toxicity caused by these medications could be prevented or counteracted by natural compounds or drugs recently developed to treat mitochondrial diseases. A recent review by Rai et al., 2016, provides an overview of a variety of pharmaceuticals which either target mitochondria or maintain its homeostasis [121]. While the majority of these drugs target diseases caused by mutations (MELAS, LHON, MERFF, etc.), one therapy is being developed for improving skeletal muscle function in elderly patients. Elamipretide (MTP-131) has been shown to prevent peroxidation of cardiolipin by binding to the molecule and preventing its interaction with cytochorme *c*, which prevents the latter from functioning as a peroxidase rather than an electron carrier. The drug is currently in Phase III [121]. Another compound is Idebenone, which is based structurally upon CoQ10. Idebanone is currently being explored for LHON and MELAS [121], but not as a supplemental drug in statin therapy. There are a host of natural products and supplements that have been discussed to improve mitochondrial function such as resveratrol, curcumin, NAD and N-acetyl cysteine (NAC), but there is no clinical evidence of clear success.

## 5. Conclusions

As patient treatment is often essential, we suggest using medication(s) with the best safety profile and the avoidance of concurrent usage of multiple medications that carry mitochondrial liabilities. Our hope is that future therapies will be devoid of mitochondrial liabilities as screening paradigms can be deployed preclinically. In addition, we also recommend lifestyle changes to further improve one’s mitochondrial function, such as weight loss, exercise and nutrition. Another major point is that the cause for mitochondrial dysfunction with age appears to be one of molecular homeostatic control of mitochondrial integrity or functional capacity, not simply an acute depletion of bioenergetic substrates, such as ATP. Consequently, bolus supplementation with mitochondrial cofactors has less potential than long-term lifestyle changes in extending vitality and drug tolerance in aging populations.

## Figures and Tables

**Table 1:** Each drug class contains drugs with more and less observed mitochondrial toxicity.

Drug Class	Rank order of Toxicity Observed (High to Low)	Target Organ
Anti-diabetic (thiazolidinediones)	Trovan * (troglitazone), Avandia (rosiglitazone), Actos (pioglitazone)	Liver [53]
Cholesterol lowering (statins)	Baycol * (cerivastatin), Zocor (simvastatin), Lipitor (atorvastatin), Lescol (fluvastatin)	Muscle [53]
Anti-diabetic (biguanides)	Phenformin * (N-phenethylbiguanide), Buformin *(1-butylbiguanide), Glucophage (metformin)	Lactic acidosis [55]
Anti-depressant/anxiety (SARIs)	Zerzone * (nefazodone), Desyrel (trazodone), Buspar (buspirone)	Liver [54]
Anti-lipidemic (fibrates)	Lopid (gemfibrozil), Lipanor (ciprofibrate), Trilepix (fenofibrate)	Liver [53]
Pain medication (NSAIDs)	Avalanche * (celebrex), Mobic (meloxicam), Voltaran (dichlofenac), Felden (piroxicam), Aspirin (acetylsalicylic acid)	Liver, intestine [56]
Antibiotics (fluoroquinolones)	Trovan * (trovafloxicin), Levaquin (levafloxicin), Cetraxal (ciprofloxicin)	Liver [57]
Anti-cancer (topoisomerase inhibitors)	Adriamycin (doxorubicin)	Heart [58,59,60,61]

* withdrawn from the market.

**Table 2:** Most Commonly Used Prescription and Over-the-Counter Medications (OTC) in US Older Adults. Modified from [64].

Medication	Brand Name/Drug Name	% Prescribed	References	Mitochondrial Toxicity Reported
Pain medication (OTC)	Aspirin (acetylsalisylic acid)	40	[65,66,67,68,69,70]	Inhibition of Respiration, Uncoupling of Oxidative Phosphorylation, Opening of MPT Pore, Inhibition of ATPase, Alteration of Glutathione Status
Cholesterol lowering	Zocor (simvastatin)	22	[53,71,72,73,74,75,76,77]	Inhibition of Respiration, Uncoupling of oxidative Phosphorylation, Inhibition of ETC Complexes I, IV, V, Decrease in Membrane Potential, Increase Ca^++^ Release, Decrease ATP Levels
Blood pressure medication	Zestril (lisinopril)	20	No reports	
Diuretic	Microzide (hydrochlorothiazidine)	19	No reports	
Thyroid medication	Synthroid (levothyroxin)	15	No reports	
Heart medication	Lopressor (metoprolol)	15	No reports	
Heartburn (OTC)	Prilosec (omeprazole)	10	[78]	Inhibition of Carnitine/Acylcarnitie Transporter
Cholesterol lowering	Lipitor (atorvastatin)	9	[79,80,81]	Inhibits Mitochondrial Respiration in Pancreatic, Cardiomyocytes and Endothelial Cells
Pain medication (OTC)	Tylenol (acetaminophen)	9	[82,83,84,85]	Opening of MPT Pore, Formation of Reactive Oxygen Species, Depletion of mtDNA
Heart medication	Tenormin (atenolol)	8	No reports	
Diuretic	Lasix (furosemide)	7	[86,87,88]	Inhibition of Respiration, Uncoupling of Oxidative Phosphorylation
Blood thinner	Plavix (clopidogrel)	7	[89,90,91]	Inhibition of Respiration, Depletion of Glutathione, Induction of Oxidative Stress, Reduction in Membrane Potential
Blood thinner	Coumadin (warfarin)	6	No reports	
Heart medication	Coreg (carvedilol)	5	[92]	Mixed Reports of Mitochondrial Toxicity vs. Preventive Mode
Cholesterol lowering	Pravachol (pravastatin)	5	[93]	Opening MPT Pore, Inhibition of Respiration at High Concentrations
Cholesterol lowering	Crestor (rosuvastatin)	5	[94]	Mixed Reports of Mitochondrial Toxicity vs. Preventive Mode
Pain medication (OTC)	Aleve (naproxen)	5	[95,96]	Inhibition of Respiration, Inhibition of Ca^++^ Flux
Cholesterol lowering	Zetia (ezetimibe)	5	No reports	

**Table 3:** Comparison of two geriatric people.

Variable	Patient 1	Patient 2
Age	75	75
Body Mass Index	18	28
Breakfast	Yogurt with granola, fresh fruit, poached egg, green tea	Eggs, bacon and potatoes
Exercise	1 h walk daily, swimming once a week	From the car to the house, around the grocery store
Lunch	Hummus and vegetables, banana, oat meal cookie	French fries and hot dogs, ice cream sundae
Medications	None	Zocor, Lopid, Prilosec, Voltaren (oral), Lasix, Nexteron
Dinner	Wild caught salmon and salad	Pasta with sausage and cheese
Drink Consumption	Red wine	Coke
